# Influence of psychosocial adaptation on existential distress among young breast cancer survivors in the rehabilitation period: An analysis of the chain mediating effects of social support and psychological resilience

**DOI:** 10.1371/journal.pone.0354635

**Published:** 2026-08-11

**Authors:** Lin Tao, Mitao Li, Jieying Lv, Qiuzhou Wang, Lan Fu, Junying Li

**Affiliations:** 1 Cancer Day-care Unit, Division of Medical Oncology, Cancer Center, West China Hospital, Sichuan University/West China School of Nursing, Sichuan University, Chengdu, China; 2 Breast Center, West China Hospital, Sichuan University/ Department of General Surgery, West China Hospital, Sichuan University/West China School of Nursing, Sichuan University, Chengdu, China; 3 Thoracic Oncology Ward, Cancer Center, West China Hospital, Sichuan University/West China School of Nursing, Sichuan University, Chengdu, China; University Center of Paulinia - UNIFACP, BRAZIL

## Abstract

This study investigated the influence of psychosocial adaptation on existential distress among young breast cancer survivors during the rehabilitation period, with a focus on the chain-mediating roles of social support and psychological resilience. A cross-sectional survey was conducted with 390 young Chinese breast cancer survivors (aged 18−49 years, within 5 years post-treatment) recruited from tertiary hospitals. Participants completed standardized measures assessing psychosocial adaptation (PAIS-SR), social support (PSSS), psychological resilience (CD-RISC10), and existential distress (DS-II). Results from correlation analysis revealed that poorer psychosocial adaptation was significantly associated with higher existential distress (r = 0.510), lower social support (r = −0.306), and reduced psychological resilience (r = −0.412). Structural equation modeling demonstrated a significant direct effect of psychosocial adaptation on existential distress (effect = 0.514). Crucially, mediation analysis using bootstrapping confirmed three significant indirect pathways: the independent mediation of social support (effect = 0.061, 51.3%), the independent mediation of psychological resilience (effect = 0.040, 33.6%), and their sequential chain mediation (effect = 0.018,15.1%). The findings indicate that social support and psychological resilience function as both independent and sequential protective factors, mitigating the impact of psychosocial adaptation challenges on existential distress. The study underscores the importance of integrated interventions that enhance external support systems and foster internal resilience to improve psychological outcomes for young breast cancer survivors navigating the unique challenges of the rehabilitation phase.

## Introduction

Breast cancer remains the most prevalent malignant tumor among women globally. Approximately 7–10% of diagnosed cases occur in women aged < 40 years [1]. Following standardized treatments, including surgery, radiotherapy, and chemotherapy, this demographic confronts physical impairments, such as breast loss and ovarian dysfunction, and navigates multifaceted psychosocial challenges, including disrupted career trajectories, derailed marital and reproductive plans, and marginalization of social roles [1,2]. Empirical evidence indicates that patients with breast cancer aged <50 years exhibit higher rates of psychological distress than do their older counterparts [3], with this psychological burden being particularly pronounced during the rehabilitation phase. Robbeson et al. [4] reported that 46.67% of breast cancer survivors experience significant psychological distress during rehabilitation. Furthermore, Zhang et al. [5] indicated that 22.5% of young and middle-aged patients exhibit persistent severe maladjustment, 50.4% demonstrate moderate maladjustment, and only 27.1% achieve optimal adaptation during the postoperative recovery period. These findings underscore the complex psychological pressures that young patients with breast cancer face during physical rehabilitation and social role reconstitution.

As a distinct population characterized by the dual burdens of “cancer trauma” and “critical life-stage development,” the relationship between psychosocial adaptation and existential distress among young breast cancer survivors in rehabilitation has garnered attention in psycho-oncology research. Psychosocial adaptation is defined as an individual’s capacity to modulate responses to physiological, psychological, and environmental changes during disease recovery [6], whereas existential distress encompasses disease-induced negative affect (e.g., anxiety and depression) and loss of meaning in life [7]. Although existing studies have established associations between psychosocial adaptation and existential distress [8], they are predominantly limited to univariate analyses and fail to examine interactive mechanisms between “external support and internal traits,” particularly disregarding the unique influence of the rehabilitation stage.

As an external protective resource, social support may serve as a mediator between psychosocial adaptation and existential distress. Prior research has demonstrated that emotional (e.g., companionship) and informational (e.g., medical literacy) support from familial, peer, and institutional sources buffer the psychological impact of disease stress [9]. For young survivors, specialized social support, such as fertility preservation counseling and vocational rehabilitation, directly mitigates role loss-related adjustment disorders, reducing existential distress. For example, spousal support exhibits a significant negative correlation with survivor distress levels [10]. Psychological resilience, an internal protective trait, may mediate the effects of psychosocial adaptation on existential distress. Individuals with high resilience are more inclined to employ adaptive coping strategies (e.g., cognitive reappraisal), reframing the disease experience as a catalyst for self-growth and alleviating negative affect [11]. Social support and psychological resilience may form a hierarchical mediational chain: social support enhances psychological resilience via self-efficacy reinforcement, buffering existential distress.

Within China’s unique cultural framework, young patients with breast cancer face compounded challenges during psychosocial adaptation. Confucian ideals of familial responsibility and social role expectations impose the psychological burden of “illness stigma,” alongside physical trauma during rehabilitation [12]. The relationship-centric ethos of collectivist cultures amplifies the salience of social support systems in psychological adjustment [13]. Concurrently, the traditional life-course imperatives of “family establishment and career development” clash with disease-induced fertility impairment and career disruption, exacerbating existential distress [14]. In China, the provision of post-treatment rehabilitation for breast cancer survivors is guided by multidisciplinary clinical recommendations and is predominantly coordinated within tertiary hospitals. These services encompass physical rehabilitation, psychological support, and long-term follow-up care [15]. However, the accessibility and continuity of such comprehensive rehabilitation are not uniform. Empirical data suggest that only an estimated 47.2% of young breast cancer survivors engage in structured, institution-based rehabilitation programs [16]. Although these existing programs typically address core needs such as physical function retraining, symptom management, and general psychological counseling, they often lack systematic, evidence-based interventions tailored to profound existential distress and multifaceted psychosocial adaptation challenges. The present study aims to address this specific gap in care by investigating the mechanisms underlying these underexplored aspects of recovery [17].

Based on the above premises, this study proposed the following hypotheses: H1: Psychosocial adaptation significantly influences existential distress among young breast cancer survivors during rehabilitation. H2: Social support mediates the relationship between psychosocial adaptation and existential distress. H3: Psychological resilience mediates the relationship between psychosocial adaptation and existential distress. H4: Social support and psychological resilience form a serial mediational pathway in the relationship between psychosocial adaptation and existential distress. This study aims to investigate the relationship between psychosocial adaptation and existential distress among young breast cancer survivors during the rehabilitation period and examine whether social support and psychological resilience mediate this relationship independently and sequentially.

## Methods

### Study design

This was a multicenter, cross-sectional study conducted between October 6, 2024, and March 30, 2025.

### Sampling and recruitment

This study recruited participants via convenience sampling from the follow‑up registries of six tertiary hospitals in Sichuan Province, China. The sites comprised one major comprehensive cancer center and five alliance-affiliated tertiary general hospitals, all of which are officially designated as provincial or regional cancer centers. Each hospital provided a standard postoperative rehabilitation protocol that typically included lymphoedema management, physical therapy, and basic psychological counseling. Written informed consent was obtained from all participants before enrollment. As no minors were included in this study, parental or guardian consent was not required.

### Inclusion and exclusion criteria

The included participants were 1) female patients pathologically diagnosed with breast cancer (Stages I–III); 2) those who had completed primary treatments such as surgery, chemotherapy, or radiotherapy, and were in the rehabilitation period (≤5 years after treatment completion); 3) those aged 18–49 years; 4) individuals with normal mental status, capable of normal communication, and having normal reading comprehension ability; and 5) those who were aware of their condition and provided informed consent. The excluded individuals were 1) patients with metastatic or recurrent breast cancer (to avoid interference of disease severity with psychological status); 2) those with other malignant tumors or severe physical diseases; 3) individuals diagnosed with mental illnesses (such as severe depression and schizophrenia) or those who had recently taken antipsychotic medications; 4) those with cognitive impairments or those unable to complete the questionnaire; and 5) patients currently receiving active antitumor treatments such as chemotherapy and radiotherapy (to avoid confounding effects from treatment side effects). In this cross-sectional study, existential distress scores served as the primary indicator. The sample size was estimated using the confidence interval (CI) for the mean. According to the pre-experiment results, the mean existential distress score was 18.72 points, with a standard deviation of 7.14. Setting α at 0.05 (two-sided Z = 1.96) and the allowable error at 0.8 points, the initial sample size was calculated by the formula n = (Z × σ/d)², resulting in n = (1.96 × 7.14/0.8)² = 306. Considering a data loss rate of 20%, the final required sample size was 306/0.8 = 383.

### Procedure

An online questionnaire was used to collect data. The researchers first obtained approval from the administrative departments of the target hospitals and recruited data collectors. All collectors underwent unified training before the formal survey to ensure the full comprehension of the research objectives, inclusion/exclusion criteria, and questionnaire administration protocols. The training content included standardized guidelines for using the Wenjuanxing platform, uniform instructional scripts, and contingency plans for unexpected situations. After ethical review and approval by the hospital ethics committee, data collectors at each hospital screened eligible patients (pathologically diagnosed with stage I–III breast cancer, aged 18–49 years, within 5 years post-treatment completion) using the electronic medical record system. Nurses explained the purpose of the study to patients during telephone follow-ups or outpatient visits, and upon obtaining consent, sent electronic questionnaire links embedded with informed consent forms. The questionnaire was secured with response passwords (hospital code + patient ID), and data quality was ensured by monitoring response times (≥ 10 min). Participants completed the e-questionnaire via mobile QR code scanning, with the system automatically validating missing items and prompting completion. Researchers conducted daily data verification, followed by telephone interviews to confirm outliers (e.g., 10 consecutive identical responses). A total of 387 valid questionnaires were retrieved, implying a response rate of 92.6%. Harman’s single-factor test indicated no significant common method bias (unrotated first factor explained variance = 18.3%). All participants received gifts as compensation for the time invested in completing the questionnaire.

## Measures

### Psychosocial adaptation

We used the Chinese version of the Psychosocial Adjustment to Illness Scale (PAIS-SR) [18]. This 44-item scale comprises seven dimensions: Health Care (seven items), Work Competence (eight items), Family Relations (seven items), Recreation (six items), Social Interaction (five items), Sexual Functioning (four items), and Emotional State (seven items). Items adopted a diversified single-response scoring system with content-specific (scoring rules), using a 4-point Likert scale (0–3). Total scores range from 0 to 132, with high values indicating pronounced psychosocial adaptation problems. The scale demonstrated good reliability (Cronbach’s α = 0.872, split-half reliability = 0.753) and structural validity in previous studies. In this study, the overall Cronbach’s α for PAIS-SR was 0.863.

### Psychological resilience

Psychological resilience was measured using the Chinese Connor-Davidson Resilience Scale (CD-RISC10) [19]. This 10-item instrument employs a 5-point Likert scale (0–4), yielding a total score of 0–40. High scores indicate great resilience. The scale’s excellent psychometric properties (Cronbach’s α = 0.902) in the original validation were replicated in this study, confirming its reliability and validity for young breast cancer survivors.

### Social support

The Perceived Social Support Scale (PSSS), originally developed by Dahlem et al. [20] and adapted by Jiang et al.[21] was used in this study. The scale comprises 12 items across three dimensions: Family Support (4 items), Friend Support (4 items), and Significant Other Support (4 items). All items were rated on a 7-point Likert scale ranging from 1 (strongly disagree) to 7 (strongly agree). Total scores range from 12 to 84, with higher scores representing greater perceived social support. The scale demonstrated good reliability in the present study (Cronbach’s α = 0.900) and has been well-validated across diverse populations. Notably, the PSSS assesses only interpersonal social support from family, friends, and significant others. It does not include professional counseling, psychological therapy, support groups, or other formal health-related services.

### Existential distress

Existential distress was assessed using the Chinese Demoralization Scale-Ⅱ (DS-Ⅱ) adapted by Ou et al. [22]. This 16-item self-report scale contains two dimensions: Meaning & Purpose and Distress & Coping Ability, using a 3-point Likert scale (0 = “never,” 1 = “sometimes,” 2 = “often”). Total scores (0–32) correlate with severity of distress, with higher scores indicating severe demoralization. The Chinese DS-Ⅱ demonstrated strong reliability (Cronbach’s α = 0.877) and content validity (scale level CVI = 0.94) in this study.

### Data analysis

Statistical analyses were conducted using SPSS 23.0 and AMOS 23.0 software. First, Pearson correlation coefficients were computed to examine the bivariate relationships among psychosocial adaptation, social support, psychological resilience, and existential distress. Structural equation modeling (SEM) was then performed to test the hypothesized associations between these variables.In the proposed model, psychosocial adaptation was specified as the independent variable, with social support and psychological resilience serving as sequential mediators, and existential distress as the outcome variable. The hypothesized serial mediation effect posited that psychosocial adaptation influences existential distress through a sequential pathway involving social support and psychological resilience.A bootstrapping procedure with 1000 resamples was used to estimate the mediation effects, with the significance level set at α = 0.05. Multiple fit indices were adopted to evaluate model fit, including the chi-square-to-degrees-of-freedom ratio (χ²/df < 3 indicates acceptable fit), goodness-of-fit index (GFI > 0.90), adjusted goodness-of-fit index (AGFI > 0.90), comparative fit index (CFI > 0.90), incremental fit index (IFI > 0.90), Tucker-Lewis index (TLI > 0.90), and standardized root mean square residual (SRMR < 0.08). Missing data accounted for less than 5% of the total dataset, and mean imputation was applied to address missing values.

### Ethical statement

The Clinical Trial and Biomedical Ethics Committee of West China Hospital, Sichuan University (No. 2024(1330)) approved the data collection procedures involving the study participants, ensuring they were conducted in accordance with the ethical standards.

## Results

### Descriptive statistics

A total of 420 potentially eligible participants were initially identified. Following preliminary screening, 405 individuals underwent eligibility assessment, of whom 398 were confirmed eligible based on strict criteria. Ultimately, 390 participants were enrolled in the study. All enrolled individuals completed the follow-up and were included in the final statistical analysis.The demographic and clinical characteristics of the participants are presented in Table 1. The study participants were predominantly aged 40–49 years (48.7%), with higher education as the most common educational level (37.7%). The majority were married (55.6%) and cohabiting (92.3%), and most had one child (39.7%). A monthly per capita family income of less than 300 USD was the most prevalent (35.9%), and part-time employment (52.6%) was more common than full-time employment(47.4%). Regarding clinical characteristics, most patients were at tumor stage II (38.5%), with a disease duration of 1–3 years since diagnosis (41.0%), and the majority had no tumor metastasis (79.5%).

**Table 1:** Descriptive statistics for sociodemographic and clinical characteristics (N = 390).

Variable	n	%	Variable	n	%
Age range (years)			Monthly family income per capita (USD)		
18–29	75	19.2	< 300	140	35.9
30–39	125	32.1	300–600	118	30.3
40–49	190	48.7	601–900	85	21.8
**Education**			901–1200	47	12.0
Primary school	45	11.5	**Employment**		
Middle school	70	17.9	Full-time job	185	47.4
Senior school	128	32.8	Part-time job	205	52.6
College or above	147	37.7	**Stage of tumor**		
**Marital status**			Tis	18	4.6
Unmarried	99	25.3	I	75	19.2
Married	217	55.6	II	150	38.5
Divorced	74	19.1	Ⅲ	147	37.7
**Living status**			**Time since diagnosis (years)**		
Living alone	30	7.7	< 1	100	25.6
Cohabitating	360	92.3	1–3	160	41.0
**Number of children**			3–5	130	33.3
0	140	35.9	**Metastasis status**		
1	155	39.7	Yes	80	20.5
≥2	95	24.4	No	310	79.5

The mean values for the variables are as follows: psychosocial adaptation, 76.41; social support, 61.15; psychological resilience, 26.35; and existential distress, 17.80 (Table 2).

**Table 2:** Descriptive statistics for psychosocial adaptation, psychological resilience, social support, and existential distress.

Variable	Mean	Standard deviation
Psychosocial adaptation	76.41	15.38
Social support	61.15	12.42
Psychological resilience	26.35	10.52
Existential distress	17.80	6.45

### Correlation analysis of each variable

As presented in Table 3, Pearson’s correlation analysis demonstrated significant associations among the variables. Psychosocial adaptation exhibited a strong negative correlation with psychological resilience (r = −0.412) and social support (r = −0.306), but a pronounced positive correlation with existential distress (r = 0.510). Psychological resilience was positively associated with social support (r = 0.419) and inversely associated with existential distress (r = −0.424). Similarly, social support showed a modest negative correlation with existential distress (r= − .319).

**Table 3:** Pearson’s correlation analysis.

Variable	Psychosocial adaptation	Psychological resilience	Social support	Existential distress
Psychosocial adaptation	1			
Psychological resilience	−0.412**	1		
Social support	−0.306**	0.419**	1	
Existential distress	0.510**	−0.424**	−0.319**	1

**P < 0.01.

### Model test and mediation analysis

**Fig 1:**
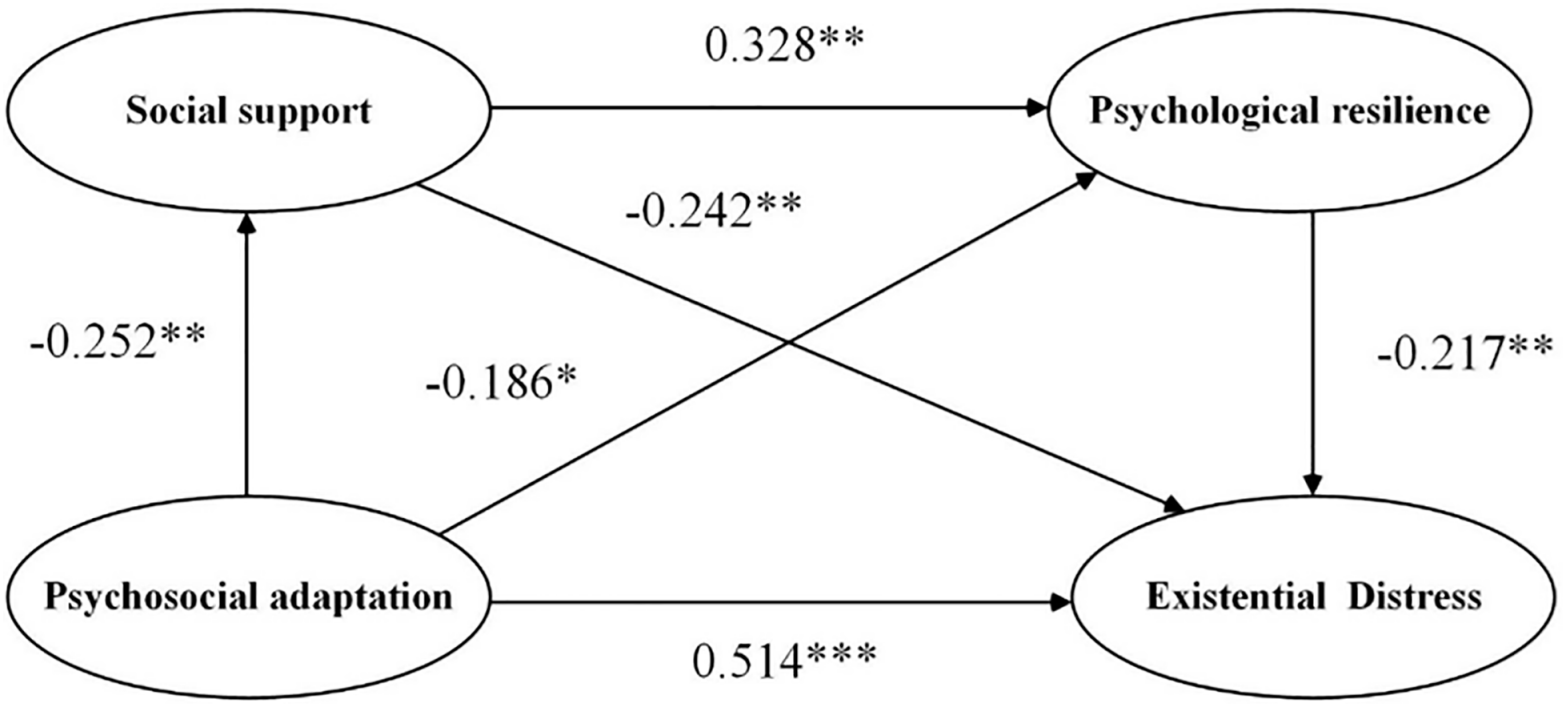
Results of the correlational model of psychosocial adaptation, psychological resilience, social support, and existential distress. **P < 0.05,**P < 0.01, ***P < 0.001.

The structural equation modeling analysis was conducted to examine the relationships among psychosocial adaptation, psychological resilience, social support, and existential distress (Fig 1). The model exhibited a good fit (χ² = 42.150 (df = 28), χ²/df = 1.505; AGFI = 0.940, GFI = 0.960, CFI = 0.958, RMSEA = 0.042). An examination of standardized path coefficients (Table 4 and the path diagram) showed that low psychosocial adaptation was linked to decreased social support (β = −0.252, CR = −2.52, p < 0.01), reduced psychological resilience (β = −0.186, CR = −1.69, p < 0.05), and high existential distress (β = 0.514, CR = 5.41, p < 0.001). Social support was positively associated with psychological resilience (β = 0.328, CR = 2.73, p < 0.01) and negatively associated with existential distress (β = −0.242, CR = −2.85, p < 0.01). High psychological resilience was associated with lower existential distress (β = −0.217, CR = −2.63, p < 0.01).

**Table 4:** Standardized estimation of each path in the modified model.

Path			Path coefficient	SE	CR	P
Psychosocial adaptation	→	Social support	−0.252	0.100	−2.52	< 0.01
Psychosocial adaptation	→	Psychological resilience	−0.186	0.110	−1.69	< 0.05
Psychosocial adaptation	→	Existential distress	0.514	0.095	5.41	< 0.001
Social support	→	Psychological resilience	0.328	0.120	2.73	< 0.01
Social support	→	Existential distress	−0.242	0.085	−2.85	< 0.01
Psychological resilience	→	Existential distress	−0.217	0.102	−2.63	< 0.01

Note. CR, critical ratio; SE, standard error.

Mediation analysis via bias-corrected bootstrapping (5,000 resamples) revealed three significant indirect pathways (Table 5). First, social support mediated the association between psychosocial adaptation and existential distress (indirect effect = 0.061, 95% CI [0.032, 0.089], accounting for 51.3% of the total indirect effect). Second, psychological resilience mediated this relationship (indirect effect = 0.040, 95% CI [0.015, 0.065], or 33.6% of the total indirect effect). Third, a sequential mediation pathway was found, in which the influence of psychosocial adaptation on existential distress operated through a sequence of social support and psychological resilience (indirect effect = 0.018, 95% CI [0.005, 0.031], 15.1% of the total indirect effect). The overall indirect effect was 0.119 (95% CI [0.090, 0.230], p < 0.05), suggesting that social support and psychological resilience act as independent and sequential protective factors in mitigating existential distress.

**Table 5:** Bootstrap intermediate effect test.

Mediation path	Estimate	Proportion of the mediation effect	95%CI		P
			Lower	Upper	
Overall indirect effect	0.119	-	0.090	0.230	< 0.05
Psychosocial adaptation → Social support→ Existential distress	0.061	51.3%	0.032	0.089	< 0.05
Psychosocial adaptation → Psychological resilience→ Existential distress	0.040	33.6%	0.015	0.065	< 0.05
Psychosocial adaptation →Social support →Psychological resilience→- Existential distress	0.018	15.1%	0.005	0.031	< 0.05

## Discussion

This study revealed a significant positive correlation between psychosocial adaptation and existential distress (including negative emotions such as anxiety and depression) among young breast cancer survivors during rehabilitation. High levels of psychosocial adaptation challenges were associated with severe existential distress, and these challenges positively predicted it, validating Hypothesis H1. This finding is consistent with those of a previous study on cancer survivors [8,23]. Young patients undergoing rehabilitation face multiple stressors, including physical functional impairments (e.g., postoperative body image changes and chemotherapy sequelae), social role transitions (e.g., work reintegration pressure and family responsibility conflicts), and fear of disease recurrence. Inadequate psychological adaptation makes these individuals more prone to self-identity confusion, social withdrawal, and loss of existential meaning [24]. Compared with the status of older patients, young individuals are in critical life stages, such as career development and marriage/childbearing; therefore, cancer diagnosis and treatment impose more severe impacts on their social functions and psychological states. For example, treatment-induced fertility impairment may trigger long-term depression, and uncertainty about future life could exacerbate anxiety [25].

Additionally, environmental changes during rehabilitation (e.g., transition from hospital treatment to home-based recovery) may lead to adjustment disorders in emotions and behaviors among young survivors, such as gaps between ideal and actual physical recovery and biases in perceiving social support, aggravating existential distress, and affecting quality of life and rehabilitation progress [26]. Therefore, monitoring the psychosocial adaptation status of young breast cancer survivors during rehabilitation and implementing timely interventions to improve their adaptive capacities represent critical strategies for reducing existential distress. Owing to the cross-sectional design of this study, causal inferences cannot be drawn from the present results; the observed associations only reflect concurrent relationships among variables.

This study confirms that social support, as a critical external coping resource, plays a significant mediating role in the relationship between psychosocial adaptation and existential distress among young breast cancer survivors during rehabilitation, thereby validating Hypothesis H2 [27]. This finding aligns with established evidence in broader cancer populations, where social support is known to buffer disease-related stress through emotional (e.g., companionship, empathy) and informational (e.g., access to medical knowledge) pathways [28]. In this specific cohort, the protective mechanism manifests through unique, developmentally sensitive pathways; the efficient utilization of support closely aligns with acute adaptation needs, particularly in fertility preservation and vocational role reconstruction. Targeted support from partners, family, and social networks directly mitigates adjustment disorders stemming from such role disruptions [27]. Clarifying that the social support measured herein refers specifically to perceived interpersonal support is crucial. Although professional counseling and structured support groups constitute valuable, distinct categories of “supportive services” for managing existential distress, they were not captured by our measure. This distinction underscores that strengthening informal, day-to-day interpersonal networks is a foundational and accessible strategy for alleviating distress in this population [26,28]. Consequently, support-based interventions should be precisely tailored to address core developmental needs, such as fertility concerns and career reintegration, and strategically integrate digital peer platforms with family-system approaches to maximize the mediating role of interpersonal support in improving psychosocial adaptation and reducing existential distress [29].

Psychological resilience significantly mediated the relationship between psychosocial adaptation and existential distress, supporting Hypothesis H3. As an internal protective factor against adversity, psychological resilience in young breast cancer survivors manifests as “bounce-back ability” from cancer trauma [30]. Individuals with high resilience actively reconstruct disease meaning (e.g., framing cancer as an opportunity for self-growth) and effectively regulate negative emotions, whereas increased psychosocial adaptation challenges weaken resilience, exacerbating existential distress. This aligns with Luo et al.‘s theoretical analysis of psychological resilience, which indicates that individual-environment interactions influence mental health through processes such as cognitive restructuring and emotional regulation [31]. For example, young survivors in rehabilitation who adopt a growth mindset toward physical changes (e.g., accepting postoperative appearance and exploring new self-expression methods) buffer the emotional impact of adjustment disorders using their psychological resilience [32]. Furthermore, the strong cognitive flexibility of young individuals implies that interventions to enhance psychological resilience (e.g., mindfulness training and social role reconstruction counseling) directly reduce the severity of existential distress symptoms such as anxiety and depression.

The chain-mediating effect of social support through psychological resilience significantly influenced the psychosocial adaptation mechanism of young breast cancer survivors (supporting H4), deepening the understanding of the collaborative role of psychosocial resources in breast cancer rehabilitation. Social support provides direct emotional comfort and practical assistance, and fosters sustained psychosocial adaptation by gradually cultivating individual psychological resilience, including enhancing self-efficacy, promoting cognitive restructuring, and improving coping skills [33]. In young patients, this chain effect may exhibit unique generational characteristics. Young patients with breast cancer prefer to exchange anticancer experiences with their peers through online platforms such as WeChat and Weibo [26,34]. Such online peer mutual aid usually builds confidence and courage to overcome disease more effectively than family care does.

Additionally, dynamic changes across disease stages moderate this mediating mechanism, and social support may primarily serve as immediate emotional buffering during the acute treatment phase while systematically transforming into psychological resilience factors during the rehabilitation maintenance phase. Individual differences significantly influence this transformation. High levels of education and excellent digital literacy facilitate the effective conversion of online social support resources into psychological resilience, a phenomenon particularly prominent in young patients. This may be related to their greater proficiency in using digital technologies to access and integrate social support. Future research should examine the differential effects of digital versus traditional social support on psychological resilience and optimize social support intervention strategies for young patients with diverse characteristics.

### Study limitations

This study provides valuable insights into the psychosocial dynamics of young breast cancer survivors. However, several methodological aspects necessitate cautious interpretation of the findings and clearly delineate the boundaries of their applicability.

First, the cross-sectional design precludes any causal inference regarding the observed relationships. Although the structural equation model demonstrated a good fit for the proposed chain-mediation pathways, the data are correlational and collected at a single time point. Therefore, the directions of the paths (e.g., from psychosocial adaptation to existential distress via social support) are grounded in theory and prior longitudinal evidence but have not been empirically proven. The associations reflect concurrent relationships, and reverse or bidirectional causality cannot be ruled out. Consequently, longitudinal studies with multiple assessment points (e.g., tracking survivors at intervals from 6 to 24 months post-treatment) are essential to empirically test the temporal sequence, causal direction, and stability of the proposed psychosocial pathways over time.

Second, while the sample size was calculated to meet statistical criteria, the convenience sampling method employed in this study is a form of non‑probability sampling, which may limit the generalizability of both the findings and the constructed model. Our results are better understood as illustrating the psychosocial relationships among factors within the specific clinical cohort studied, rather than being directly generalizable to all young breast cancer survivors across the nation.Moreover, this study was conducted in Sichuan Province. Participants’ psychological experiences, the social support they received, and the healthcare services they accessed are all embedded in the local cultural and medical context, which is further shaped by broader Chinese cultural norms such as collectivism and family roles. Therefore, caution is warranted when extrapolating the conclusions to other regions or countries with different socio-medical systems. Under different cultural and institutional settings, the patterns and strength of associations among variables may also vary.

Third, the exclusion of patients with metastatic or recurrent disease, while methodologically justified to ensure a homogeneous rehabilitation cohort, narrows the clinical scope of the conclusions. Existential distress is often more profound and complex in the context of advanced or progressive illness. By focusing on non-metastatic survivors, this study likely captures a less severe spectrum of such distress. Consequently, our findings and the resulting intervention implications may not directly apply to the subgroup of patients with advanced disease, who may face distinct and more severe psychosocial adaptation challenges.

Fourth, the reliance on self-reported measures carries inherent limitations. Although we employed validated scales and statistical tests (e.g., Harman’s single-factor test), which suggested that common method bias was not severe, it cannot be eliminated. Potential biases such as social desirability (e.g., underreporting of distress) or recall inaccuracy might have influenced the correlations among variables.

Fifth, the study did not account for several potential confounding variables, such as specific personality traits (e.g., neuroticism, optimism), detailed treatment modalities and their side-effect profiles, or genetic factors. The omission of these variables leaves open the possibility of confounding, and the unique contributions of psychosocial adaptation, social support, and resilience to existential distress may be partially attributable to other unmeasured factors.

## Conclusion

This study elucidates the complex interplay between psychosocial adaptation and existential distress among young breast cancer survivors during rehabilitation, highlighting the mediating roles of social support and psychological resilience. The findings demonstrate that psychosocial adaptation challenges significantly predict high existential distress, whereas social support and psychological resilience serve as independent and sequential buffers against such distress. The chain-mediation pathway underscores the significance of integrating external support systems with internal resilience-building strategies in psychological interventions. These results align with and extend prior research by emphasizing the unique challenges faced by young survivors, such as fertility concerns and career disruption, which necessitate targeted support. Addressing these psychosocial dynamics may help healthcare providers mitigate existential distress and enhance the overall well-being of this vulnerable population.

### Relevance to clinical practice

This study has crucial implications for clinical practice in psycho-oncology. First, healthcare providers should routinely assess psychosocial adaptation and existential distress in young breast cancer survivors during rehabilitation using validated tools, such as the PAIS-SR and DS-II. The early identification of high-risk individuals may facilitate timely interventions. Second, interventions should prioritize strengthening informal social support networks (e.g., family, partners, and peers), particularly by addressing group-specific needs such as fertility counseling and vocational rehabilitation. Additionally, facilitating access to formal supportive services (e.g., professional psychological counseling and structured support groups) should be considered a complementary strategy, as these address needs that may extend beyond the scope of interpersonal support. Third, resilience-building strategies (e.g., cognitive-behavioral therapy and mindfulness training) should be integrated into rehabilitation plans to help patients reframe adversity and enhance their coping skills. Finally, a multidisciplinary approach involving oncologists, psychologists, and social workers is essential to address the multifaceted challenges young survivors face. Thus, clinicians can foster adaptive recovery and improve long-term psychological outcomes by leveraging external and internal resilience.
